# Responses of holocyclic and anholocyclic *Rhopalosiphum padi* populations to low‐temperature and short‐photoperiod induction

**DOI:** 10.1002/ece3.2720

**Published:** 2017-01-19

**Authors:** Xiong Peng, Xianfeng Qiao, Maohua Chen

**Affiliations:** ^1^ Northwest A&F UniversityYanglingShaanxi ProvinceChina; ^2^ State Key Laboratory of Crop Stress Biology for Arid AreasKey Laboratory of Crop Pest Integrated Pest Management on the Loess Plateau of Ministry of AgricultureYanglingShaanxi ProvinceChina

**Keywords:** cost of sex, gynoparae, life cycle, life table, male, oviparae

## Abstract

The different life cycles of aphid species make these organisms good models for studying the short‐term consequences of sex. The bird cherry‐oat aphid *Rhopalosiphum padi* has a wide geographic distribution and correspondingly different life cycles. In this study, the life cycles of *R. padi* collected from six different regions in China were characterized experimentally by comparing the responses of holocyclic and anholocyclic populations to low‐temperature and short‐photoperiod induction. Clones collected from Chuzhou, Taian, and Taigu consistently reproduced via obligate parthenogenesis, whereas clones from Hami and Baicheng were holocyclic in their response, and those from Lanzhou were both holocyclic and anholocyclic. Prolonged exposure to low temperature and a short photoperiod (LS) had negative effects on the offspring of anholocyclic aphids with regard to adult lifespan, total longevity, and fecundity compared with aphids maintained at a normal temperature and a long photoperiod (NL). Holocyclic LS *R. padi* had longer developmental times at all nymph stages, a shorter adult lifespan, shorter total longevity, and a lower fecundity than NL counterparts. The adult prereproduction period of gynoparae was significantly longer than that of virginoparae, and the total longevity of gynoparae was significantly shorter than that of virginoparae. Moreover, the net reproductive and gross reproduction rates, as well as the total fecundity, were roughly fivefold higher in virginoparae than in gynoparae, indicating that there is the short‐term cost of sex. When maintained on their secondary host (*Triticum aestivum*), gynoparae, males, and oviparae produced by holocyclic populations could survive, and gynoparae produced oviparae. However, under NL conditions, oviparae could not produce overwintering eggs on the secondary host, whereas a few overwintering eggs were generated by oviparae under LS conditions. Taken together, these results illuminate the complexity of insect responses and contribute to a complete understanding of the aphid life cycle and its evolution.

## 1 Introduction

Two much‐debated issues in evolutionary biology concern the long‐term persistence and the origin of sex and asexuality in organisms. In general, asexual lineages have many evolutionary disadvantages because their offspring are of identical genotype, and favorable mutations, by‐products of meiosis, are not possible, whereas deleterious mutations can easily accumulate (Dixon, 1985; Gilabert et al., 2009). Thus, at least theoretically, asexual lineages should ultimately be eliminated (Hadany & Beker, 2003; Muller, 1964). Sexual reproduction, by contrast, can reduce the accumulation of deleterious mutations, a process referred to as Muller's ratchet (Muller, 1964), and play a role in the progressive changes in telomere length (Lushai & Loxdale, 2007). However, in addition to the ability of asexual populations to very rapidly outcompete sexual ones under some conditions, asexuality may be advantageous because of the twofold cost of sex (the cost of males and of passing on only half of the individual's genes to offspring). The many ecologically and genetically based theories and hypotheses addressing the paradox of sex and its long‐ and short‐term advantages (Butlin, 2002; Kondrashov, 1994; Muller, 1964) offer a conceptual framework for studying geographic parthenogenesis (Law & Crespi, 2002).

Among insects, aphids provide an excellent model for studying the evolution of sex, empirically examining the relative costs and benefits of sexual versus asexual reproduction, and identifying the ecological cost of switching from one to the other. A holocyclic life cycle with cyclical parthenogenesis, in which many parthenogenetic generations alternate with a single annual sexual generation, is one of the most remarkable polyphenisms in aphids. The sexual phase of cyclical parthenogenesis is triggered by environmental changes, with photoperiod, which acts as the main seasonal cue, and temperature the most crucial factors (Lees, 1966). Aphids can assess the time still available to produce appropriate aphid forms based on the progress of the photoperiod (Nylin & Gotthard, 1998). Temperature often interacts with photoperiod and modifies its effect. The sexual morphs of aphids can be induced by the appropriate combination of a short photoperiod together and a low temperature (Simon, Blackman, & Le Gallic, 1991). The mating of sexual females and male aphids results in overwintering eggs, which are more resistant than adults to harsh conditions, such as freezing, which typically kill asexual forms (Nespolo, Halkett, Figueroa, Plantegenest, & Simon, 2009; Rispe, Simon, & Pierre, 1996). The link between sexuality and cold resistance can be seen as a contingent short‐term advantage for sex. Conversely, the aphid sexual phase can also be facultative or lost by some genotypes. Anholocyclic aphid lineages with obligate parthenogenesis reproduce parthenogenetically during the whole year and, despite the appropriate inducing conditions, do not produce sexual morphs.

The bird cherry‐oat aphid *Rhopalosiphum padi* (L.), one of the most globally abundant cereal aphid pests, reproduces through cyclical or obligate parthenogenesis. Clones capable of cyclical parthenogenesis persist over many asexual generations during the summer on species that act as their secondary hosts (Poaceae) but form a single sexual generation on their primary host (*Prunus* L.) (Rispe, Bonhomme, & Simon, 1999; Simon et al., 1991). Empirical studies have identified differences in the responses of *R. padi* from different regions to a short photoperiod and low temperature (Delmotte, Leterme, Gauthier, Rispe, & Simon, 2002; Duan, Peng, Qiao, & Chen, 2016; Halkett, Plantegenest, Bonhomme, & Simon, 2008; Hulle, Maurice, Rispe, & Simon, 1999; Simon et al., 1996). Intraspecific variation in reproductive strategy has also been studied in other species of the family Aphididae, including *Myzus persicae* Sulzer (Blackman, 1974; Guillemaud, Mieuzet, & Simon, 2003; Margaritopoulos, Tsitsipis, Goudoudaki, & Blackman, 2002; Vorburger, Sunnucks, & Ward, 2003), *Aphis gossypii* (Fuller, Chavigny, Lapchin, & Vanlerberghe‐Masutti, 1999; Razmjou, Vorburger, Moharramipour, Mirhoseini, & Fathipour, 2010; Slosser, Pinchak, & Rummel, 1989; Stoetzel, Miller, O'Brien, & Graves, 1996), *Acyrthosiphon pisum* (Kanbe & Akimoto, 2009), and *Sitobion avenae* (Dedryver, Hullé, Le Gallic, Caillaud, & Simon, 2001; Simon et al., 1999).

Besides reproductive polyphenisms, organisms have other life‐history traits, including development time, fecundity, survival rate, and longevity, that are influenced by natural selection and may be constrained by genetic factors that are differentially expressed depending on the specific conditions (Homeny & Juliano, 2007). In nature, adaptation to the spatial heterogeneity of the environment results in intraspecific geographic variations in relevant ecophysiological traits (Dmitriew, 2011; Rajpurohit, Nedvěd, & Gibbs, 2013). Lineage specialization governed by genetic factors is also a trait of aphids, including the relative investment in sexual reproduction, which may be influenced by energy constraints in a given environment. The relative investment in all forms of reproduction will determine the life‐history strategy of an aphid lineage and, therefore, also its fitness (Stearns, 1989).

In autumn, male and gynoparous females of *R. padi* parthenogenetically produced by virginoparae on secondary hosts (Poaceae) fly to their primary host (*Prunus* L.). There, the gynoparous females parthenogenetically produce oviparous (sexual) females that are able to mate with males, resulting in the production by oviparae of cold‐resistant, diapausing eggs. However, in the absence of the primary host, questions about whether gynoparae and males can survive on the secondary host, whether the gynoparae can produce oviparae on this host, and whether oviparae can then produce cold‐resistant eggs remain unanswered. By contrast, lineages that undergo obligate parthenogenesis do so continuously with viviparous females throughout the year. Despite the numerous studies that have investigated the reproductive strategies of *R. padi* from different regions and evaluated the short‐term costs and benefits of the different reproductive modes, direct measurements of the life‐history traits of *R. padi* from different regions and the quantitative parameters of sexual versus asexual morphs under normal rearing conditions (24°C and a light:dark cycle of 16:8 hr) have yet to be determined.

In this study, we examined the influence of a prolonged exposure to low temperature and a short photoperiod on the reproduction of *R. padi* from different regions. Additionally, we investigated the life history of gynoparae, males, and oviparae on their secondary host. Our aim was to analyze the reproductive modes of *R. padi* from different regions and thus to elucidate the effects of low temperature and a short photoperiod on *R. padi* populations with anholocyclic and holocyclic life cycles. The survival and reproduction of sexual forms maintained on the secondary host were also investigated. Our results contribute to a better understanding of life‐cycle evolution in aphids.

## 2 Materials and Methods

### 2.1 Experimental organisms

Six *R. padi* populations collected from different geographic regions including Baicheng of Jilin Province (coordinates: 45°39′N, 122°52′E; altitude: 149 m; the sample was coded as JB), Hami of Xinjiang Province (43°34′N, 93°21′E; 1,795 m; XH), Lanzhou of Gansu Province (36°05′N, 103°41′E; 1537 m; GL), Taigu of Shanxi Province (37°25′N, 112°34′E; 798 m; STG), Taian of Shangdong Province (36°06′N, 117°14′E; 126 m; ST), and Chuzhou of Anhui Province (32°21′N, 118°20′E; 27 m; AC). The sampling time was variable because of the variability in the peak period of *R. padi* occurrence, reflecting differences in agricultural cultivation periods and climatic conditions. At least 100 wingless adults were collected from several cultivated wheat plants at each location and transported back to the laboratory. These individuals were used to initiate a separate laboratory colony representative of the region of origin; the colony was referred to as a single population. To minimize the chance of resampling individuals from the same parthenogenetic mother, each wingless adult aphid was collected from plants separated from one another by at least 20 m. Prior to the experiment, all colonies were reared on *T. aestivum* seedlings for three generations in cultures maintained at 24°C, 70% relative humidity, and a L:D cycle of 16:8 hr to eliminate maternal and grand‐maternal environmental effects from the clonal lineages (Pitchers et al., 2013; Zhang, Qiao, & Peng, 2016).

### 2.2 Determination of the life cycles of *Rhopalosiphum padi*

To confirm the modes of reproduction of *R. padi* from different regions, the life cycles of 15 individuals from each area were examined. The aphids were exposed to a short photoperiod (8‐hr:16‐hr L:D cycle) and low temperature (12°C) (Blackman, Malarky, Margaritopoulos, & Tsitsipis, 2007; Dedryver, Le Gallic, Gauthier, & Simon, 1998; Delmotte, Leterme, Bonhomme, Rispe, & Simon, 2001; Margaritopoulos et al., 2002; Simon et al., 1991) in a program‐controlled incubator to determine their reproductive mode.

To initiate the experiment, 15 second‐instar nymphs from each area raised under normal conditions were selected, transferred to the inducing conditions, and monitored until they reached adulthood (G_0_). They were then allowed to reproduce; 3 days later, the adults were removed from the plants and again reared under normal conditions. The aphid forms were identified using an anatomical microscope when the G_1_ individuals became adults. At the same time, winged parthenogenetic females and gynoparae were reared separately to observe the offspring morphs, because they could not be discriminated clearly based on their morphological characteristics alone. G_1_ individuals were allowed to reproduce for only 1 day because colonies reared at high density preferably give rise to winged aphids. The same process was followed to ascertain aphid morphs of the G_2_–G_5_ generations. The reproductive mode of a clone was determined according to its ability to produce different aphid forms during six generations of sex induction.

### 2.3 Comparison of the life‐history traits of anholocyclic and holocyclic *Rhopalosiphum padi*

After the determination of *R. padi* populations from different regions, at least 100 wingless adults per population were randomly chosen and the aphids from each population were reared separately. Following larviposition by these wingless adults, the newly born nymphs of each population were randomly divided into two groups. The NL group was maintained under normal temperature and a long photoperiod, whereas the LS group was maintained under a low temperature and a short photoperiod. Newly born nymphs of the NL group were maintained in environmental growth chambers at 24°C and a photoperiod of 16‐hr:8‐hr L:D cycle (normal conditions), and those of the LS group were maintained under the inducing conditions of a constant short photoperiod of 8‐hr:16‐hr L:D cycle at 12°C. After being fed for two generations, the apterous one‐day‐old adult aphids of the second generations (G_2_) of groups NL and LS were transferred to caged host plants, where they were allowed to reproduce for 2 hr, after which they were removed from the plant to obtain cohorts of same‐aged first‐instar nymphs of *R. padi*. The cohort of each population was reared in a program‐controlled incubator at 24°C, a relative humidity of 70%, and a photoperiod of 16‐hr:8‐hr L:D cycle to obtain a life table for each population. Only one newborn aphid nymph was placed in each of the prepared seedlings, which were used at the three‐leaf stage. To minimize the effect of the nutritional conditions provided by the host plants, the wheat seedlings were replenished every 7 days. For trait measurements, the test aphid was monitored twice daily, and molting and mortality were recorded at about the same time each day. During the reproductive period of adults, newborn nymphs were counted and removed twice daily. This process was continued until all the adult aphids died.

### 2.4 Comparison of the life‐history traits of the sexual generation under normal and inducing conditions

To examine the survivorship and reproduction of males and oviparae on the secondary host (*T. aestivum*), 10 aphids were randomly collected from each of the three holocyclic colonies and then used to establish a clone. These 30 clones were raised separately under a constant short photoperiod of 8‐hr:16‐hr L:D cycle and a temperature of 12°C (inducing conditions) for three generations to obtain a sexual generation. Adult gynoparae and virginoparae of the third generation (G_3_) from each clone were then randomly divided into two groups, one of which was maintained under a constant long photoperiod of 16‐hr:8‐hr L:D cycle at 24°C, and the other of which was maintained under low temperature (12°C) and a short photoperiod (8‐hr:16‐hr L:D cycle). The adult gynoparae and virginoparae of each group were allowed to reproduce for 12 hr on *T. aestivum*, after which they were removed from the plants. The offspring of these gynoparae and virginoparae were identified as males, oviparae, or gynoparae according to their morphological characteristics after they had molted into their final adult form (Duan et al., 2016; Simon, Rispe, & Sunnucks, 2002). The identified males and oviparae were then randomly divided into two groups; one group (~20 aphids per rearing condition) was still reared individually to observe the life history of each aphid using the same methods described for the life tables; the other group (~30 aphids per rearing condition) was used to observe reproduction on the secondary host (*T. aestivum*). Five males and five oviparae of the latter group were placed together for mating and egg laying in a breeding cage containing *T. aestivum* seedlings. The breeding cage was a transparent cubic Plexiglas container (10 × 10 × 10 cm) with the top replaced by a fine mesh gauze cover. A piece of white filter paper was placed above the nutritive medium of the seedlings to catch any eggs that fell. The number of eggs was counted 10 days later.

### 2.5 Life tables and statistical analysis

The following traits were measured in both the NL and the LS groups according to the method of Birch (1948): age‐specific survival rate (*l*_x_), longevity of each aphid, age‐stage‐specific survival rate (*s*_xj_), and age‐specific fecundity (*m*_x_). From these data, the mean generation time (*T*), reproductive value (*V*_x_), intrinsic rate of increase (*r*_m_), net reproductive rates (*R*_0_), mean fecundity, age‐stage life expectancy (*e*_xj_), gross reproductive rate (GRR), and finite rate of increase (λ) were calculated using the TWO SEX‐MSChart program (Chi, 2005).

The significance of the differences in the life‐table parameters of group NL versus group LS was analyzed using a one‐way ANOVA test. For parameters determined to be significantly different (*p *< .05), the mean values of the two groups were then compared using the least significant difference (LSD) test (SAS Institute, 2003). Data of the survival rates and the percentages of the various aphid forms were log‐transformed to meet the assumptions of normality and homoscedasticity required for these analyses. Then, the means were compared using the least significant difference (LSD) test (*p *< .05) when the overall variation in ANOVA was significant. All statistical analyses were performed using the SAS software (SAS Institute 2003).

## 3 Results

### 3.1 The reproductive mode of *Rhopalosiphum padi* from six populations

The percentages of aphid forms of clones collected from different regions and maintained under a low temperature and short photoperiod are shown in Table 1. Among the five generations, only alate and apterous parthenogenetic females, not sexuale (oviparae and males), were produced by clones collected in Taigu (Shanxi Province), Taian (Shandong Province), and Chuzhou (Anhui Province) (Table 1), indicating that the life cycle of *R. padi* from these regions is anholocyclic.

**Table 1:** Percentages (mean ± *SE*) of five different aphid forms in *Rhopalosiphum padi* from six different regions under inducing conditions

Populations (no. of clones)	Aphid forms	Generations					Life cycles
		G_1_	G_2_	G_3_	G_4_	G_5_	
JB‐CP (*n* = 15)	Gynoparae	0	29.65 ± 3.40	36.99 ± 0.98	24.08 ± 1.54	7.25 ± 2.13	Holocyclic
	Male	0	1.10 ± 0.33	24.31 ± 1.04	26.44 ± 1.08	16.70 ± 2.79	
	Oviparae	0	0	19.57 ± 1.29	41.84 ± 1.85	76.05 ± 4.35	
	Alatae virginoparae	16.02 ± 5.86	5.35 ± 0.56	2.22 ± 0.63	0	0	
	Apterae virginoparae	83.98 ± 5.86	63.90 ± 3.84	16.91 ± 1.13	7.64 ± 1.50	0	
XH‐CP (*n* = 15)	Gynoparae	0	39.79 ± 2.08	42.42 ± 1.15	31.51 ± 0.82	14.29 ± 1.51	Holocyclic
	Male	0	0.25 ± 0.25	16.28 ± 0.56	23.39 ± 0.67	29.63 ± 0.99	
	Oviparae	0	0	27.85 ± 1.08	40.16 ± 0.85	56.08 ± 1.76	
	Alatae virginoparae	7.35 ± 2.85	24.59 ± 1.62	5.49 ± 0.84	0	0	
	Apterae virginoparae	92.65 ± 2.85	35.37 ± 2.16	7.96 ± 0.97	4.94 ± 0.32	0	
GL‐CP (*n* = 7)	Gynoparae	0	9.10 ± 2.55	30.98 ± 3.72	30.99 ± 3.23	17.07 ± 2.77	Holocyclic
	Male	0	0.33 ± 0.33	13.77 ± 1.41	21.13 ± 1.03	34.33 ± 1.88	
	Oviparae	0	0	32.31 ± 3.21	43.57 ± 3.29	48.60 ± 1.33	
	Alatae virginoparae	0	9.23 ± 3.34	9.27 ± 2.97	0	0	
	Apterae virginoparae	100	81.34 ± 5.80	13.67 ± 3.19	4.31 ± 1.69	0	
GL‐OP (*n* = 8)	Gynoparae	0	0	0	0	0	Anholocyclic
	Male	0	0	0	0	0	
	Alatae virginoparae	1.14 ± 1.13	4.06 ± 1.25	23.10 ± 4.66	20.22 ± 4.25	30.42 ± 5.73	
	Apterae virginoparae	98.86 ± 1.13	95.94 ± 1.25	76.90 ± 4.66	79.78 ± 4.25	69.58 ± 5.73	
	Oviparae	0	0	0	0	0	
STG‐OP (*n* = 15)	Gynoparae	0	0	0	0	0	Anholocyclic
	Male	0	0	0	0	0	
	Oviparae	0	0	0	0	0	
	Alatae virginoparae	4.26 ± 2.31	45.10 ± 4.31	23.42 ± 4.74	14.85 ± 0.88	22.33 ± 3.98	
	Apterae virginoparae	95.74 ± 2.31	54.90 ± 4.31	76.58 ± 4.74	85.15 ± 0.88	77.67 ± 3.98	
ST‐OP (*n* = 15)	Gynoparae	0	0	0	0	0	Anholocyclic
	Male	0	0	0	0	0	
	Oviparae	0	0	0	0	0	
	Alatae virginoparae	56.92 ± 6.84	9.78 ± 1.71	18.95 ± 6.78	20.06 ± 3.58	22.05 ± 3.02	
	Apterae virginoparae	43.08 ± 6.84	90.22 ± 1.71	81.05 ± 6.78	79.94 ± 3.58	77.95 ± 3.02	
AC‐OP (*n* = 15)	Gynoparae	0	0	0	0	0	Anholocyclic
	Male	0	0	0	0	0	
	Oviparae	0	0	0	0	0	
	Alatae virginoparae	0.44 ± 0.44	10.08 ± 2.22	15.04 ± 2.41	14.26 ± 2.62	31.91 ± 4.31	
	Apterae virginoparae	99.56 ± 0.44	89.92 ± 2.22	84.96 ± 2.41	85.74 ± 2.62	68.09 ± 4.31	

JB‐CP, the cyclic parthenogenetic clones from Baicheng; XH‐CP, the cyclic parthenogenetic clones from Hami; GL‐CP and GL‐OP, the cyclic parthenogenetic and the obligate parthenogenetic clones from Lanzhou; STG‐OP, the obligate parthenogenetic clones from Taigu; ST‐OP, the obligate parthenogenetic clones from Taian; AC‐OP, the obligate parthenogenetic clones from Chuzhou. G_1_–G_5_ indicate the first, the second, the third, the fourth, and the fifth generations, respectively.

A holocyclic response consists of distinct reproductive periods. Gynoparae are produced for the first time in the second generation, and their proportion of the total population reaches a peak in the third generation, after which it steadily declines. Males are produced by very few clones in the second generation, but the proportion increases thereafter and reaches a peak in the fifth generation. Oviparae are first produced in the third generation, after which production is similar to that of males. Alate and apterous parthenogenetic females are no longer produced in the fifth generation, respectively (Table 1). The clones from Baicheng of Jilin Province and Hami of Xinjiang Province were able to produce gynoparae, males, and ovipare and thus exhibited holocyclic life cycles.

Among the clones sampled from Lanzhou, eight produced only alate and apterous parthenogenetic females, not sexuale. Accordingly, these eight clones had an anholocyclic life cycle. Another seven clones produced only gynoparae, males, and oviparae (Table 1) and therefore also had holocyclic life cycles. Clones sampled from Lanzhou had a mixture of holocyclic and anholocyclic life cycles. Two subpopulations from Lanzhou, consisting of obligate parthenogenetic (GL‐OP) and cyclic parthenogenetic (GL‐CP) clones, were distinguished (Table 1).

### 3.2 Comparisons of the effect of inducing conditions on the developmental period, adult longevity, total longevity, fecundity, and nymph mortality of anholocyclic and holocyclic populations

Among the four anholocyclic populations, the developmental time of first‐instar nymphs (Table 2; ANOVA: *F *= 29.30; *df* = 1, 318; *p *< .001) of *R. padi* was shorter in group LS than in group NL, whereas the developmental times of second‐instar (*F *= 1.71; *df* = 1, 318; *p *= .19), third‐instar (*F *= 0.40; *df *= 1, 314; *p *= .53), and fourth‐instar (*F *= 0.68; *df *= 1, 309; *p *= .41) nymphs from the two groups were not significantly different. The effects of the prolonged exposure of anholocyclic populations to inducing conditions were significant on total adult lifespan (*F *= 11.67; *df *= 1, 300; *p *< .001) and total longevity (*F *= 12.50; *df *= 1, 318; *p *< .001). The mean fecundity of group LS was significantly lower than that of group NL (*F *= 12.50; *df *= 1, 300; *p *< .001), whereas the difference in nymph mortality was not significant (*F *= 1.04; *df *= 1, 6; *p *= .35).

**Table 2:** Comparisons of the effect of inducing conditions to developmental periods, adult longevity, total longevity, fecundity, and nymph mortality of anholocyclic and holocyclic populations in *Rhopalosiphum padi* (mean ± *SE*)

Populations	Life‐history traits	Group NL	Group LS	*df*	*F* value	*p* Value
Anholocyclic populations	L1	1.42 ± 0.026	1.20 ± 0.027	1, 318	29.30	<.001
	L2	1.17 ± 0.022	1.21 ± 0.021	1, 318	1.71	.19
	L3	1.11 ± 0.019	1.10 ± 0.016	1, 314	0.40	.53
	L4	1.30 ± 0.022	1.27 ± 0.024	1, 309	0.68	.41
	Adult longevity	14.05 ± 0.47	11.76 ± 0.48	1, 300	11.67	<.001
	Total nymph stage	5.00 ± 0.044	4.82 ± 0.042	1, 300	9.41	.0024
	Total longevity	18.39 ± 0.52	15.81 ± 0.51	1, 318	12.50	<.001
	Fecundity	62.99 ± 1.54	54.48 ± 1.86	1, 300	12.50	<.001
	Nymph mortality (%)	4.38 ± 1.20	6.88 ± 2.14	1, 6	1.04	.35
Holocyclic populations	L1	1.40 ± 0.025	1.48 ± 0.015	1, 238	7.23	.0077
	L2	1.29 ± 0.032	1.55 ± 0.025	1, 238	38.78	<.001
	L3	1.12 ± 0.020	1.49 ± 0.033	1, 238	88.35	<.001
	L4	1.16 ± 0.028	1.49 ± 0.038	1, 233	48.86	<.001
	Adult longevity	17.63 ± 0.42	10.85 ± 0.39	1, 217	137.74	<.001
	Total nymph stage	4.96 ± 0.052	6.07 ± 0.077	1, 217	148.50	<.001
	Total longevity	21.84 ± 0.52	15.36 ± 0.51	1, 238	78.90	<.001
	Fecundity	67.71 ± 1.28	18.82 ± 1.92	1, 207	477.87	<.001
	Nymph mortality (%)	4.17 ± 3.00	13.33 ± 4.64	1, 4	2.75	.17

L1–L4 represents the developmental period of the first‐, the second‐, the third‐, and the fourth‐instar nymph stages, respectively; NL, the population which was fed in normal temperature and long photoperiod conditions; LS, the population which were induced by low temperature and short photoperiod conditions; *p* value of LSD test is shown in the last column.

Among the three holocyclic populations, the developmental time of group LS nymphs was significantly longer than that of group NL nymphs (Table 2; first‐instar nymphs: *F *= 7.23; *df *= 1, 238; *p *= .0077; second‐instar nymphs: *F *= 38.78; *df *= 1, 238; *p *< .001; third‐instar nymphs: *F *= 88.35; *df *= 1, 238; *p *< .001; fourth‐instar nymphs: *F *= 48.86; *df *= 1, 233; *p *< .001; total nymph stage: *F *= 148.50; *df *= 1, 217; *p *< .001). Total adult lifespan (*F *= 137.74; *df *= 1, 217; *p *< .001) and total longevity (*F *= 78.90; *df *= 1, 238; *p *< .001) were significantly shorter, and mean fecundity was significantly lower (*F *= 477.87; *df *= 1, 207; *p *< .001) in group LS than in group NL. By contrast, nymph mortality did not significantly differ between the two groups (Table 2; *F *= 2.75; *df *= 1, 4; *p *= .17).

### 3.3 The effect of inducing conditions on the life‐table parameters of anholocyclic and holocyclic populations

The effects of the inducing conditions on the intrinsic rate of increase (*r*), finite rate of increase (λ), mean generation time (*T*), net reproductive rate (*R*_0_), and gross reproduction rate (GRR) of the *R. padi* populations from six regions are summarized in Table 3. Overall, the values of all five parameters differed significantly between groups NL and LS. In holocyclic populations, four values (*r*, λ, *R*_0_, and GRR) were significantly higher in group NL than in group LS, whereas the mean generation time (*T*) of group LS was longer than that of group NL. Among the four anholocyclic populations, only the net reproduction rate was significantly higher in group NL than in group LS; no similar trends were detected in the other life‐table parameters. However, in group LS, the values of the four life‐table parameters (*r*, λ, *R*_0_, and GRR) were significantly higher in anholocyclic than in holocyclic *R. padi*, whereas the latter had a longer mean generation time.

**Table 3:** Comparisons of the effect of inducing conditions to life‐table parameters of *Rhopalosiphum padi* from six regions (mean ± *SE*)

Parameters	Groups	Populations, mean ± *SE*						
		JB‐CP	XH‐CP	GL‐CP	GL‐OP	STG‐OP	ST‐OP	AC‐OP
Intrinsic rate of increase (*r*)	NL	0.52 ± 0.0098^a^	0.50 ± 0.0065^a^	0.49 ± 0.010^a^	0.48 ± 0.0054^b^	0.51 ± 0.011^a^	0.47 ± 0.0098^b^	0.50 ± 0.0069^a^
	LS	0.28 ± 0.019^b^	0.26 ± 0.016^b^	0.30 ± 0.028^b^	0.51 ± 0.0096^a^	0.47 ± 0.012^b^	0.54 ± 0.0089^a^	0.44 ± 0.012^b^
Finite rate of increase (λ)	NL	1.68 ± 0.017^a^	1.65 ± 0.011^a^	1.63 ± 0.017^a^	1.62 ± 0.0087^b^	1.66 ± 0.017^a^	1.60 ± 0.016^b^	1.65 ± 0.011^a^
	LS	1.32 ± 0.025^b^	1.29 ± 0.020^b^	1.34 ± 0.037^b^	1.66 ± 0.016^a^	1.61 ± 0.019^b^	1.72 ± 0.015^a^	1.56 ± 0.019^b^
Net reproduction rate (Ro)	NL	65.30 ± 3.28^a^	64.65 ± 1.33^a^	64.71 ± 3.76^a^	63.40 ± 3.42^a^	59.45 ± 3.89^a^	61.90 ± 3.23^a^	58.21 ± 3.33^a^
	LS	15.39 ± 2.95^b^	14.36 ± 2.44^b^	14.48 ± 3.16^b^	60.30 ± 4.57^b^	48.11 ± 3.67^b^	57.31 ± 3.63^b^	35.48 ± 3.27^b^
Mean generation time (*T*)	NL	8.02 ± 0.10^b^	8.31 ± 0.095^b^	8.54 ± 0.12^b^	8.65 ± 0.11^a^	8.07 ± 0.13^b^	8.82 ± 0.14^a^	8.13 ± 0.095^a^
	LS	9.67 ± 0.34^a^	10.29 ± 0.24^a^	8.96 ± 0.26^a^	8.04 ± 0.12^b^	8.16 ± 0.14^a^	7.45 ± 0.084^b^	8.05 ± 0.11^b^
Gross reproduction rate (GRR)	NL	72.00 ± 2.04^a^	65.56 ± 1.03^a^	73.13 ± 1.10^a^	77.65 ± 1.59^b^	74.92 ± 1.56^a^	68.10 ± 1.27^b^	73.05 ± 1.78^a^
	LS	20.48 ± 4.32^b^	19.04 ± 3.30^b^	20.32 ± 4.01^b^	79.07 ± 1.87^a^	64.60 ± 1.55^b^	69.32 ± 1.95^a^	49.89 ± 2.30^b^

NL, the population which was fed in normal temperature and long photoperiod conditions; LS, the population which were induced by low temperature and short photoperiod conditions; The parameters on the two groups followed by different lower letters are significantly different at *p* < .05 (LSD test). JB‐CP, the cyclic parthenogenetic clones from Baicheng of Jilin Province; XH‐CP, the cyclic parthenogenetic clones from Hami of Xinjiang Province; GL‐CP and GL‐OP, the cyclic parthenogenetic and the obligate parthenogenetic clones from Lanzhou of Gansu Province, respectively; STG‐OP, the obligate parthenogenetic clones from Taigu of Shanxi Province; ST‐OP, the obligate parthenogenetic clones from Taian of Shandong Province; AC‐OP, the obligate parthenogenetic clones from Chuzhou of Anhui Province.

**Figure 1:**
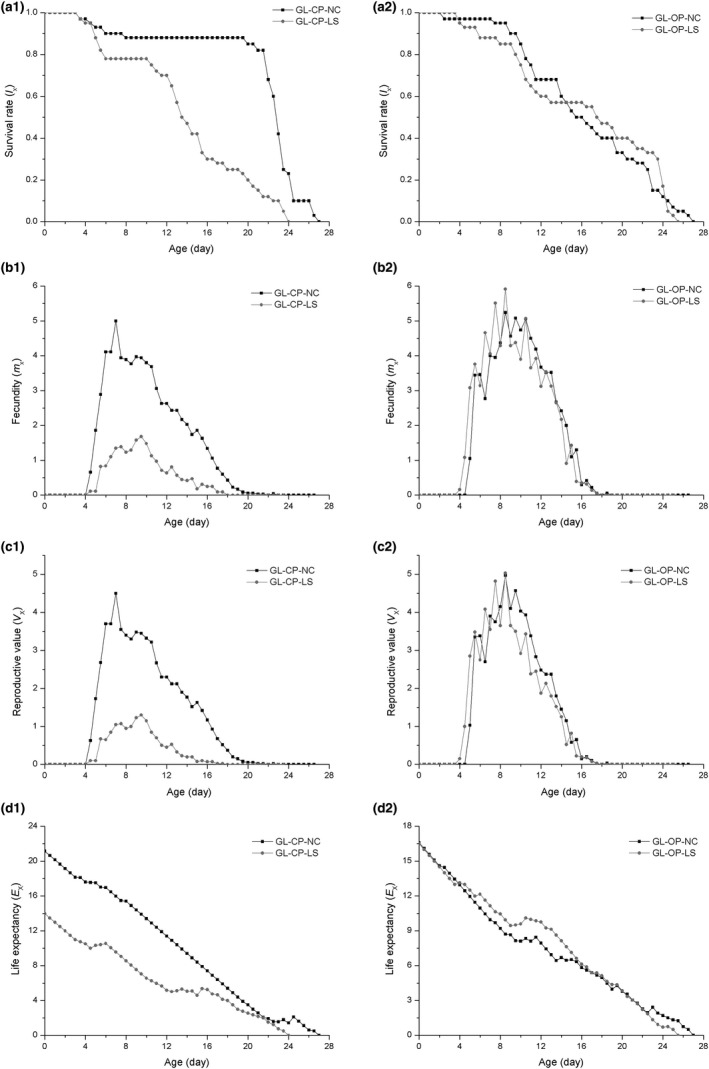
Four life‐table parameters of holocyclic and anholocyclic *Rhopalosiphum padi* populations sampled from Lanzhou. (a1–2) Age‐specific survival rate (*l*_x_); (b1–2) age‐specific fecundity (*m*_x_); (c1–2) reproductive value (*V*_x_); (d1–2) life expectancy (*E*_x_)

The two populations (GL‐OP and Gl‐CP) from the same region but with different life cycles were used to directly compare life‐table parameters, including age‐specific survival rate (*l*_x_), age‐specific fecundity (*m*_x_), reproductive value (*V*_x_), and life expectancy (*E*_x_). The results are shown in Figure 1. In the GL‐CP population, but not the GL‐OP population, the curves of survival rate, fecundity, reproductive value, life expectancy, and female fecundity differed significantly between group NL and group LS.

### 3.4 Comparison of gynoparae and virginoparae produced by three holocyclic populations

The life‐history traits of gynoparae, including the developmental time of second‐instar nymphs (*F *= 8.42; *df *= 1, 90; *p *= .0047), the adult lifespan (*F *= 121.01; *df *= 1, 90; *p *< .001), and the total longevity (*F *= 123.71; *df *= 1, 90; *p *< .001), differed significantly from those of virginoparae (Table 4). However, gynoparae and virginoparae did not significantly differ with respect to the developmental time of first‐instar nymphs (*F* = 3.77; *df* = 1, 90; *p* = .055), third‐instar nymphs (*F *= 0.83; *df *= 1, 90; *p *= .36) and fourth‐instar nymphs (*F *= 0.49; *df *= 1, 90; *p *= .49) or the age at reproduction (*F *= 0.90; *df *= 1, 90; *p *= .35). The adult prereproduction period of gynoparae (1.39 ± 0.10 days) was significantly longer than that of virginoparae (0.74 ± 0.12 days) (*F *= 11.22; *df *= 1, 90; *p *< .01).

**Table 4:** The results of one‐way ANOVA investigating the variance for the developmental periods and total longevity of *Rhopalosiphum padi* within gynoparae and virginoparae

Parameters	Source	*df*	Mean square	*F*	*p* Value
L1	Among aphid forms	1	0.11	3.77	.055
	Within	90	0.029		
L2	Among aphid forms	1	0.57	8.42	.0047
	Within	90	0.067		
L3	Among aphid forms	1	0.091	0.83	.36
	Within	90	0.11		
L4	Among aphid forms	1	0.044	0.49	.49
	Within	90	0.091		
Adult	Among aphid forms	1	841.75	121.01	<.001
	Within	90	6.96		
Total longevity	Among aphid forms	1	936.86	123.71	<.001
	Within	90	7.57		
Prereproduction period of adult	Among aphid forms	1	7.34	11.22	.0012
	Within	90	0.65		
Age at reproduction	Among aphid forms	1	1.24	0.90	.35
	Within	90	1.38		

L1–L4 represents the developmental period of the first‐, the second‐, the third‐, and the fourth‐instar nymph stages, respectively; *p* value of LSD test is shown in the last column.

The major life‐table parameters of gynoparae and virginoparae produced by the three holocyclic populations are summarized in Table 5. Overall, the intrinsic rate of increase, finite rate of increase, fecundity, net reproductive rate, and gross reproduction rate varied significantly among the three populations. Interestingly, the values of these same parameters were significantly higher in virginoparae than in gynoparae at each location. The difference in the net reproductive rate, gross reproduction rate, and total fecundity was roughly fivefold higher in the GL‐CP than in the GL‐OP population.

**Table 5:** Five life‐table parameters of gynoparae and virginoparae which were produced by holocyclic clones (mean ± *SE*)

Parameters	Forms	Locations		
		XH‐CP	JB‐CP	GL‐CP
*r*	Gynoparae	0.23 ± 0.0075^b^	0.28 ± 0.0077^b^	0.28 ± 0.019^b^
	Virginoparae	0.33 ± 0.021^a^	0.38 ± 0.041^a^	0.46 ± 0.029^a^
λ	Gynoparae	1.26 ± 0.0095^b^	1.33 ± 0.010^b^	1.32 ± 0.025^b^
	Virginoparae	1.39 ± 0.029^a^	1.47 ± 0.060^a^	1.59 ± 0.0045^a^
Ro	Gynoparae	9.38 ± 0.55^b^	12.52 ± 0.65^b^	11.05 ± 0.98^b^
	Virginoparae	32.55 ± 5.77^a^	50.47 ± 10.34^a^	57.89 ± 5.60^a^
GRR	Gynoparae	9.50 ± 0.56^b^	12.81 ± 0.62^b^	11.41 ± 0.97^b^
	Virginoparae	34.29 ± 5.44^a^	50.60 ± 10.29^a^	57.89 ± 5.60^a^
Fecundity	Gynoparae	9.38 ± 0.55^b^	12.52 ± 0.65^b^	11.05 ± 0.98^b^
	Virginoparae	32.55 ± 5.77^a^	50.47 ± 10.34^a^	57.83 ± 6.12^a^

JB‐CP, the cyclic parthenogenetic clones from Baicheng of Jilin Province; XH‐CP, the cyclic parthenogenetic clones from Hami of Xinjiang Province; GL‐CP, the cyclic parthenogenetic clones from Lanzhou of Gansu Province; *r*, intrinsic rate of increase; λ, finite rate of increase; Ro, net reproduction rate; GRR, gross reproduction rate. The parameters on the two groups followed by different lower letters are significantly different at *p* < .05 (LSD test).

### 3.5 Comparison of male and oviparae produced by three holocyclic populations under normal and inducing conditions

**Figure 2:**
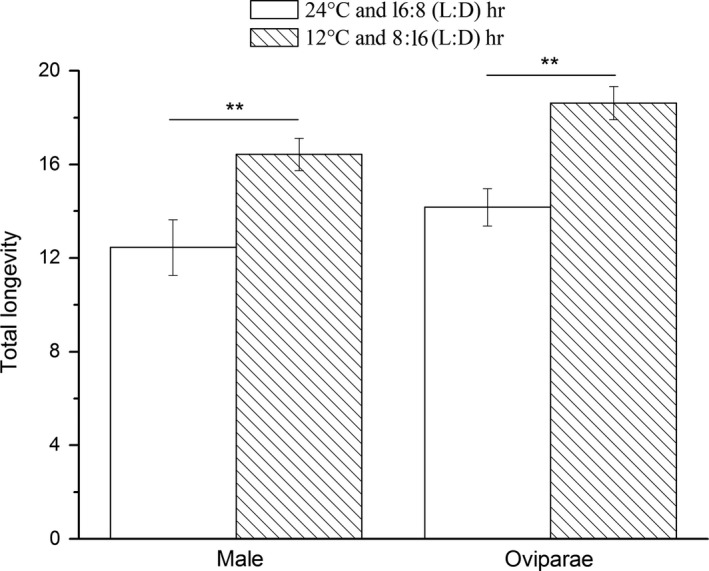
Comparisons of the total longevity (mean ± *SE*) of males and oviparae under different temperatures and photoperiods; **significant differences between populations (*p *< .01, ANOVA followed by LSD tests)

When maintained on the secondary host, males and oviparae survived and gynoparae produced oviparae. The total longevities of oviparae and males under normal conditions (24°C and a L:D cycle of 16:8 hr) were 14.17 ± 0.80 days and 12.45 ± 1.19 days, respectively, whereas the corresponding values were 18.63 ± 0.71 days and 16.43 ± 0.70 days under inducing conditions (12°C and a L:D cycle of 8:16 hr) (Figure 2). The differences in the total longevities of both oviparae (*F* = 17.34; *df* = 1, 26; *p *< .001) and males (*F* = 9.53; *df* = 1, 23; *p *< .01) feeding on the secondary host but reared under the two different conditions were significant. Additionally, although oviparae could not produce overwintering eggs on the secondary host under normal conditions, a few overwintering eggs (2.63 ± 0.41) were generated under inducing conditions.

## 4 Discussion

### 4.1 Life cycles of *Rhopalosiphum padi*

This study identified numerous polymorphisms of *R. padi* collected from different regions in China. Clones from Taigu, Taian, and Chuzhou were anholocyclic, whereas those from more northern populations (Baicheng and Hami) were holocyclic. The clones from Lanzhou, which is south of Baicheng and Hami but north of Taian and Chuzhou, exhibited a mixture of holocyclic and anholocyclic life cycles. These results suggest that *R. padi* is able to adapt to local climatic conditions by investing in two overwintering strategies, overwintering eggs and parthenogenetic females, which provide a biological advantage and maximum energy use in terms of survival and spread. The maintenance of reproductive polymorphism was discussed in an earlier study (Halkett et al., 2008). The relative frequency of different life cycles varies geographically, with the proportion of holocyclic clones declining and that of anholocyclic clones increasing along a gradient from higher to lower latitudes. Life‐cycle polymorphisms were also discovered for *R. padi* populations in Sweden (Wiktelius, 1982), the UK (Loxdale & Lushai, 2007; Tatchell, Plumb, & Carter, 1988), and France (Halkett et al., 2008; Hulle et al., 1999; Simon et al., 1996). The distribution of clones differing in their life cycles is probably maintained by powerful selective forces that prevent homogenization of the reproductive modes of different populations. These forces are predominantly ecological, mainly regional differences in winter severity (Dedryver et al., 2001; Duan et al., 2016; Halkett et al., 2004; Rispe & Pierre, 1998; Rispe, Pierre, Simon, & Gouyon, 1998; Simon et al., 1999). According to the model of Rispe et al. (1998), “sexual lineages” predominate in regions with cold winters because of the ecological advantage conferred by overwintering eggs, whereas essentially asexual lineages predominate in regions with mild winters, which support higher fecundity and fitness. Baicheng and Hami are located at relatively high latitudes, where the winters are severe, whereas Taigu, Taian, and Chuzhou are located at lower latitudes, where winters are warmer. Winter temperatures are warmer in Lanzhou than in Baicheng and Hami; however, they are much colder than in Taigu, Taian, and Chuzhou (China Meteorological database, http://data.cma.cn/), which would at least partially explain the phenotypic variations among the six populations when reared under inducing conditions.

### 4.2 The effect of long‐term inducing conditions on offspring of anholocyclic populations

Previous reports showed that holocyclic aphid clones maintained over the long term at a low temperature and short photoperiod could produce sexual generation (Dedryver et al., 2001; Rispe & Pierre, 1998; Rispe et al., 1998; Simon et al., 1999). By contrast, in this study, anholocyclic clones exposed to long‐term inducing conditions could not produce males or oviparae, and the aphid offspring had a shorter adult lifespan, shorter total longevity, and reduced fecundity. These results indicate the existence of environmentally based maternal effects, even in anholocyclic clones.

Maternal effects are defined as the non‐Mendelian influence of the maternal phenotype or environment on the phenotype or developmental characteristics of the offspring (Beckerman, Benton, Ranta, Kaitala, & Lundberg, 2002; Hunter, 2002; Mousseau & Dingle, 1991; Mousseau & Fox, 1998), and they are common in nature. Environmentally induced maternal effects have been particularly well documented across a range of insect species, including *A. pisum* (Mclean, Ferrari, & Godfray, 2009), *Aphidius ervi* (Ismaeil et al., 2013), *Aphis nerii* (Zehnder & Hunter, 2007), *Brevicoryne brassicae* (Ruiz‐Montoya & Nunez‐Farfan, 2009), *Coleomegilla maculata* (Vargas, Michaud, Nechols, & Moreno, 2014), *Drosophila serrata* (Magiafoglou & Hoffmann, 2003), *Scathophaga stercoraria* (Scharf, Bauerfeind, Blanckenhorn, & Schafer, 2010), and *S. avenae* (Jeffs & Leather, 2014). Among the most important environmental factors affecting life‐history traits and morphology are temperature and photoperiod (Scharf et al., 2010; Vaghina, Voinovich, & Reznik, 2014). The influence of temperature on the development time and longevity of insects has been described, with insects exposed to higher temperatures having a shorter life span (Ismaeil et al., 2013; Mironidis & Savopoulou‐Soultani, 2008) and those exposed to longer photoperiods having a longer developmental period, greater longevity, and increased fecundity. The age at first reproduction is also influenced by the photoperiod (Greenberg, Sappington, Adamczyk, Liu, & Setamou, 2008; Malaquias et al., 2010; Reznik & Vaghina, 2013). Maternal heat stress was also shown to negatively affect the developmental time and nymphal birthweight of the G1 progeny (Ismaeil et al., 2013; Jeffs & Leather, 2014). Our results similarly suggest that exposing aphids to a low temperature and short photoperiod negatively impacts certain life‐history traits and parameters of their progeny, mimicking the effects of seasonality.

### 4.3 Comparisons of the life‐history traits of gynoparae, virginoparae, males, and oviparae

In the autumn, gynoparae, which fly from the secondary host back to the primary host, are produced as a response to long nights and low temperatures. They differ from virginoparae in their olfactory responses at the peripheral level (Park, Elias, Donato, & Hardie, 2000) and show significantly larger electroantennogram responses to nepetalactol and nepetalactone than do alate and apterous virginoparae (Park & Hardie, 2002). This may explain the significantly reduced fecundity and shorter longevity of gynoparae than virginoparae, because of associated energy restrictions. Over the first 20 days of reproductive life, *S. avenae* virginoparae maintained under long‐day conditions were significantly more fecund than gynoparae (Newton & Dixon, 1988). In our study, the chosen measure of fitness was the intrinsic rate of increase (*r*), which is a commonly used metric in invertebrates (Carter, Simon, & Nespolo, 2012; Emery, Rice, & Stanton, 2010). We showed that the intrinsic rate of increase in virginoparae was higher than that of gynoparae, which suggested the better adaptation of the former to varying environmental conditions.

When maintained on wheat seedlings, males and oviparae did not produce overwintering eggs under normal environmental conditions (24°C and a L:D cycle of 16:8 hr), but they were able to mate with each other and produce overwintering eggs under inducing conditions (12°C and a L:D cycle of 8:16 hr). These results suggest that the long photoperiod and high temperature accounted for the absence of overwintering egg. Additionally, one of the most important extrinsic factors determining aphid fitness is the host plant species (Wool & Hales, 1997). In nature, on the primary host, wingless oviparae produced by gynoparae mate with males and produce overwintering eggs. However, diapause eggs are also generated on the secondary host. These results point to the ecological advantages of holocyclic clones. Specifically, holocyclic lineages can overwinter on the secondary host when the primary host is scarce or cannot be found by males and gynoparae.

### 4.4 The short‐term cost of sex

The survival rate of virginoparae increased, the development time decreased, and aphid fecundity increased in mild versus cold winters. The short‐term cost of sex was evidenced by the following: (1) the switch from asexual to sexual reproduction of holocyclic lineages in autumn spanned several generations; (2) the net reproductive and gross reproduction rates, as well as the total fecundity, were roughly fivefold higher in virginoparae than in gynoparae; (3) the adult prereproduction period of gynoparae was significantly longer than that of virginoparae; and (4) the total longevity of gynoparae was significantly shorter than that of virginoparae. Moreover, the longevity of males and oviparae was shorter under control than under inducing conditions, which suggests fewer opportunities for the mating of males and oviparae. Consequently, asexual lineages would have a > twofold advantage in terms of increasing their population numbers (Artacho, Figueroa, Cortes, Simon, & Nespolo, 2011).

Although cyclical parthenogenesis is the ancestral mode of aphid reproduction, these insects exhibit four distinct reproductive modes. Delmotte et al. (2001) reported that *R. padi* has multiple routes to asexuality, including the complete, spontaneous loss of sex and repeated gene flows from essentially asexual to sexual lineages. The high costs of sex under mild winter conditions might explain the complete, spontaneous loss of sex. In this study, we examined the effects of altering temperature and photoperiod on the life‐history traits of *R. padi*. Our findings will be enhanced by studies of the evolution of the life cycles of *R. padi*.

## 5 Conclusion

This study identified two different life cycles (holocyclic and anholocyclic) in six geographically distant populations of *R. padi*. Holocyclic and anholocyclic populations differed significantly with respect to their development, reproduction, and responses to low temperature and a short photoperiod. Specifically, the net reproductive rate, gross reproduction rate, and total fecundity were approximately fivefold higher in virginoparae than in gynoparae, whereas the adult prereproduction period was significantly longer in the latter. Together with the significantly shorter total longevity of gynoparae than virginoparae, our results provide evidence for the short‐term cost of sex. Interestingly, oviparae were able to survive and produce overwintering eggs on a secondary host, which demonstrated the adaptive plasticity of this life form to primary and secondary hosts. However, there is still only scant information on the genetic basis of the different life cycles and life‐history traits. Revealing the intrinsic mechanisms underlying these processes will be the next challenge in understanding the responses of *R. padi* to seasonal constraints.

## Conflict of Interest

All authors disclose any potential sources of conflict of interest.
